# Emergence of Multidrug Resistant Hypervirulent ST23 *Klebsiella pneumoniae:* Multidrug Resistant Plasmid Acquisition Drives Evolution

**DOI:** 10.3389/fcimb.2020.575289

**Published:** 2020-11-20

**Authors:** Chaitra Shankar, Jobin John Jacob, Karthick Vasudevan, Rohit Biswas, Abi Manesh, Dhiviya Prabaa Muthuirulandi Sethuvel, Santosh Varughese, Indranil Biswas, Balaji Veeraraghavan

**Affiliations:** ^1^ Department of Clinical Microbiology, Christian Medical College and Hospital, Vellore, India; ^2^ College of Biological Sciences, University of Minnesota, Saint Paul, MN, United States; ^3^ Department of Infectious Diseases, Christian Medical College and Hospital, Vellore, India; ^4^ Department of Nephrology, Christian Medical College and Hospital, Vellore, India; ^5^ Department of Microbiology, Molecular Genetics and Immunology, University of Kansas Medical Centre, Kansas City, KS, United States

**Keywords:** *Klebsiella pneumoniae*, hypervirulence plasmid, ST23, hybrid genome, multidrug resistance, OXA-232

## Abstract

**Background:**

In recent years, the emergence of multidrug resistant hypervirulent *K. pneumoniae* (MDR hvKp) isolates poses severe therapeutic challenge to global public health. The present study used the complete genome sequence of two MDR hvKp isolates belonging to ST23 to characterize the phylogenetic background and plasmid diversity.

**Methods:**

Two hvKp isolates from patients with bacteremia were sequenced using Ion Torrent PGM and Oxford Nanopore MinION platforms and assembled by hybrid genome assembly approach. Comparative genomics approaches were used to investigate the population structure, evolution, virulence, and antimicrobial resistance of MDR hvKp strains.

**Results:**

The study isolates exhibited typical features of hvKp phenotypes associated with ST23. The convergence of multidrug resistance and hypervirulence were attributed by the presence of multiple plasmids including a 216 kb virulence plasmid and MDR plasmids belonging to IncA/C_2_, IncFIB, IncX3, and ColKP3 groups. The insertion of *catA1 gene* into virulence plasmid was observed along with genetic factors such as aerobactin, salmochelin, and *rmpA2* that confer hvKp’s hypervirulent phenotype. The core genome single nucleotide polymorphism (SNP) phylogenetic analyses of the isolates showed the evolution of ST23 hvKp was predominantly driven by ICE*Kp* acquisitions.

**Conclusion:**

To the best of our knowledge, this is the first report of MDR hvKp isolates of ST23 with insertion of *catA1* gene into the virulence plasmid which presents the possibility of hotspot integration sites on the plasmids to aid acquisition of AMR genes. ST23 is no longer confined to susceptible strains of hvKp. Our findings emphasize the need for more studies on recombinant events, plasmid transmission dynamics and evolutionary process involving hvKp.

## Introduction

*Klebsiella pneumoniae* is a notorious nosocomial pathogen responsible for a wide range of healthcare associated infections and is commonly multidrug resistant (MDR). Thus, limited therapeutic options are available to control the infections caused by this pathogen (Martin and Bachman, 2018)**.** However, hypermucoviscous *K. pneumoniae*, which mostly arises from the community associated infections, retains susceptibility to antimicrobials (Liu and Guo, 2019). The hypermucoviscous pathotype also exhibits hypervirulence (hv) and manifests invasive infections by causing pyogenic liver abscesses and subsequent bacteraemia, pneumonia, meningitis, or brain abscesses (Paczosa and Mecsas, 2016). Hypervirulent *K. pneumoniae* (hvKp) strains were initially reported from Taiwan, South Korea, and other South East Asian countries however these strains became increasingly prevalent worldwide (Siu et al., 2012).

In the past few years, several hvKp strains evolved into MDR-hvKp due to the acquisition of mobile genetic elements and MDR plasmids (Lee et al., 2017). The recent reports of carbapenem resistant hvKp (CR-hvKp) carrying *bla*_KPC_ (Dong et al., 2018),*bla*_NDM_ (Roulston et al., 2018; Yuan et al., 2019), and *bla*_OXA-232_ (Shu et al., 2019) is a matter of major public health concern. The convergence of virulence with antimicrobial resistance (AMR) in hvKp isolates represent a real threat to the treatment and management of *K. pneumoniae* infections (Zhang et al., 2020). Thus, understanding the genetic background and transmission of MDR-hvKp strains from different geographical locations is an urgent priority (Wyres et al., 2019).

HvKp isolates are frequently associated with K1 and K2 capsular serotypes that facilitate the pathogen to escape from phagocytosis and intracellular killing (Shon et al., 2013; Luo et al., 2014). A combination of iron acquisition systems such as, enterobactin (*ent*), yersiniabactin (*ybt*), and *kfu* in the chromosome together with other key virulence factors such as, aerobactin, salmochelin, *rmpA*, and *rmpA2* encoded on a single large virulence plasmid (pLVPK) are associated with severe infections and a high mortality rate (Fu et al., 2019; Marr and Russo, 2019). Interestingly the *ybt* locus is mobilized by the chromosomally encoded integrative conjugative element ICE*Kp* (Guo et al., 2017; Lam et al., 2018b).

To date, there remains only a few studies to understand the genetics and transmission of MDR hvKp in India. We recently reported ST23 hvKp isolates that were susceptible to all the antimicrobials tested (Shankar et al., 2018). Our ESBL and carbapenem resistant hvKp isolates did not belong to ST23 (Shankar et al., 2016a; Shankar et al., 2016b). The pan-susceptible ST23 hvKp carries a limited number of AMR plasmids in addition to a virulence plasmid. In contrast, the present study, describes ST23 MDR hvKp that carry up to seven AMR plasmids. To the best of our knowledge, this is the first study from India characterizing ST23 multidrug hypervirulent isolates carrying *catA1*, coding for chloramphenicol resistance, on the virulence plasmid and is studied using a hybrid genome assembly combining Ion Torrent and ONT MinION technologies.

## Materials and Methods

### Bacterial Isolates

The two *K. pneumoniae* isolates included in the study were obtained from the blood culture of two patients from different wards and were collected within a period of 4 months at Christian Medical College, Vellore, India. The isolate BA4656 was isolated from a patient who had been involved in a road traffic accident and suffered from acute kidney failure and sepsis. The patient had received antibiotic treatment elsewhere before being brought to the hospital. The second isolate BA34918 was isolated from a patient who was diagnosed with advanced cholangiocarcinoma type 3A and acute cholecystitis. Bacterial isolates were identified and confirmed by VITEK-MS (Database v2.0, bioMerieux, France). Screening for the hypermucoviscous phenotype was carried out using the string test as described previously (Shon et al., 2013).

### Antimicrobial Susceptibility Testing

Antimicrobial susceptibility testing was performed for first and second line antibiotics using the Kirby-Bauer disc diffusion method (Bayer et al., 1966). The isolates were tested against ceftazidime (30 μg), cefepime (30 μg), piperacillin/tazobactam (100/10 μg), meropenem (10 μg), gentamicin (10 μg), amikacin (30 μg), ciprofloxacin (5 μg), and minocycline (30 μg). Multidrug resistance is defined as resistance to one or more antimicrobial agents in ≥ 3 classes (Magiorakos et al., 2012). *Escherichia coli* ATCC 25922, *Enterococcus* *faecium* ATCC 29212, and *Pseudomonas aeruginosa* ATCC 27853 were used as control strains for antimicrobial susceptibility testing. The minimum inhibitory concentration (MIC) of various antimicrobials was determined by VITEK2 using N281 card following the manufacturer’s protocol. Interpretation of the antimicrobial susceptibility results were done according to breakpoints defined by Clinical and Laboratory Standards Institute (CLSI) guidelines for *K. pneumoniae* (CLSI 2018). For tigecycline the MIC was determined using the broth microdilution method as per breakpoints defined by FDA.

### Whole Genome Sequencing

Total genomic DNA from the isolates was extracted from an overnight culture (14–16 h) grown at 37°C on blood agar using the fully automated QIAsymphony instrument (Qiagen, Germany) according to the manufacturer’s instructions. The extracted DNA was quantified using NanoDrop One spectrophotometry (Thermo Fisher Scientific, MA, USA) and Qubit 3.0 Fluorometry (Life Technologies, CA, USA) and stored at −20°C until further use.

The genomic DNA samples were subjected to whole genome sequencing using the Ion Torrent PGM platform with Ion 316™ chip v2 for 400bp chemistry sequencing (Life Technologies, Carlsbad, CA). For this, DNA library was prepared using 1 g of the genomic DNA using Ion Xpress Plus Fragment Library Kit (Life Technologies) following the protocol recommended for 400 bp fragment library preparation. For long read sequencing, Oxford Nanopore MinION sequencing device was used with FLO-MIN106 R9 MinION flow cells. Long read DNA library was prepared using the SQK-LSK108 ligation sequencing kit (v.R9) along with ONT EXP-NBD103 Native Barcode Expansion kit following the manufacturer’s protocol (Oxford Nanopore Technologies, Oxford, UK). The library was loaded onto the flow cells, run for nearly 48 h using the standard MinKNOW software. The Fast5 files generated from MinION sequencing were subjected to base calling with Albacore software (v.2.0.1).

### Hybrid Genome Assembly and Evaluation

Hybrid *de novo* assembly, quality check, and associated assembly statistics were performed using Ion Torrent and ONT MinION reads as described previously (Vasudevan et al., 2020). For accurate assembly the MinION long reads were error-corrected with the standalone Canu (v.1.7) using the “-correct -nanopore-raw” module (Koren et al., 2017). This was followed by hybrid *de novo* assembly using both Ion torrent and MinION reads using the Unicycler hybrid assembly pipeline (v 0.4.6) for prokaryotic genomes with the default settings (Wick et al., 2017). The complete circular genome was initially assembled and was polished with multiple rounds of Pilon (v.1.22) to reduce the base level errors as described previously (Walker et al., 2014). Genome assembly by combining the Ion Torrent short reads and the MinION long reads improved the contiguity and completeness of the genomes with fewer error rates. The quality measurements of the complete genome sequences after hybrid assembly were compared with the short reads assembled *de novo* using SPAdes v.3.12 algorithm (Bankevich et al., 2012).

To validate the quality of assembly after each polishing step the completeness, correctness and contiguity parameters were assessed using CheckM v1.0.5 (Parks et al., 2015) and Quast v4.5 (Gurevich et al., 2013). CheckM estimated the completeness and contiguity while Quast was used to detect mis-assemblies, mismatches, and indels by aligning the assemblies with the reference genome (AP006725). The genome sequences of the chromosomes and plasmids have been deposited in GenBank under the accession numbers CP035905-CP035912 and CP036190-CP036198 for BA4656 and BA34918 isolates, respectively.

### Comparative Genome Analysis

Genomes were annotated using* *Prokaryotic* *Genome Annotation Pipeline (PGAP; v.4.1) from NCBI (Tatusova et al., 2016). Genome sequences of BA4656 (NZ_CP035905) and BA34918 (NZ_CP036190) were aligned with the genome sequences of two reference isolates, SGH10 (NZ_CP02580) and NTUH-K2044 (NC_012731) using the Geneious Prime software. The genomes were rotated and reverse complemented to set the start location for all the genomes as *dnaA* gene. The genomes were aligned using Geneious Prime software with progressive Mauve algorithm. We used default parameters to automatically calculate seed weight and minimum locally collinear blocks (LCBs) score. Gap alignment was performed using MUSCLE 3.6 program.

The resistance profile of the assembled genomes was identified using ResFinder (v.3.1) on web based server available at https://cge.cbs.dtu.dk/services/ResFinder. Similarly, the presence of plasmids in the genomes were identified and characterized using PlasmidFinder (v.1.3) available at https://cge.cbs.dtu.dk/services/PlasmidFinder. Further, sequence typing of the assembled genomes was determined by the seven gene multi-locus sequence typing (MLST) scheme available at Kleborate (Lam M. et al., 2018). The K and O antigen loci of BA4656 and BA34918 were also identified using Kleborate. The genomes were mapped and analyzed against the reference strains and were visualized using CGview server v.1.0 (Grant and Stothard, 2008).

Identification of virulence factors such as yersiniabactin, aerobactin, and other siderophore production systems were carried out using Kleborate. Mobile genetic elements (MGE) in the genome sequences were identified by IS finder (https://www-is.biotoul.fr/) for insertion sequences and INTEGRALL for integrons (http://integrall.bio.ua.pt/). Two transposons were identified in the isolates, for which the accession numbers were assigned by Transposon (Tn) Registry as Tn*6691 *and Tn*6692* (https://transposon.lstmed.ac.uk/tn-registry). The ICEKp genomic island was identified using IslandViewer (Bertelli et al., 2017) and further confirmed through multiple alignment using Progressive Mauve (Darling et al., 2010), and NCBI BLAST (Johnson et al., 2008). The linear comparison of the ICEKp genomic island and the transposons were visualized using Easyfig (Sullivan et al., 2011). The presence TA systems in the strains were determine by using web-based TAfinder tool (https://db-mml.sjtu.edu.cn/TAfinder/index.php).

### Phylogenetic Analysis

The genomic sequences from two *K. pneumoniae* isolates BA4656 and BA34918 were compared to the global collection of CG23 clones. The nucleotide sequences were obtained from GenBank (https://www.ncbi.nlm.nih.gov/genbank/). The sequencing reads for previously reported 192 hypervirulent *K. pneumoniae* global isolates (Lam et al., 2018a) were downloaded from ENA (https://www.ebi.ac.uk/ena) and subsequently assembled using SPAdes v.3.12 (Bankevich et al., 2012). Furthermore, the BacWGSTdb (http://bacdb.org/BacWGSTdb/) was used to investigate the relationship between the strains (Ruan and Feng, 2016).

The genomes were mapped to the reference genome ED23 (CP016814.1) using the BWA MEM (https://github.com/lh3/bwa) algorithm and Snippy v.4.5.1 (Seemann, 2015) was used to call the genomic variants. Additionally, the variants were then filtered using FreeBayes (https://github.com/ekg/freebayes). The core SNP genome alignment of all the genomes was generated with snippy-core. The recombination regions within the core genome alignment was further filtered and removed using the Gubbins (v. 2.4.1) algorithm (Croucher et al., 2015). The maximum likelihood (ML) phylogeny was constructed using FastTree v.2.1.8 (Price et al., 2009) using GTR model with 100 bootstrap replicates. The phylogenetic tree was rooted with the reference genome and labeled using the Interactive Tree of Life software (iTOL v.3) software (Letunic and Bork, 2016).

## Results

### Phenotypic Antibiotic Resistance Profiles of Multidrug Resistant Hypervirulent *K. pneumoniae* Isolates BA4656 and BA34918

We observed both BA4656 and BA34918 strains were phenotypically resistant to all the tested antimicrobials from disc diffusion assay. The MIC values for antimicrobials as determined by VITEK2 (bioMerieux) were as follows: piperacillin/tazobactam (≥128μg/ml), cefoperazone/sulbactam (≥ 64μg/ml), cefepime (≥ 64μg/ml), ceftazidime (≥ 64μg/ml), aztreonam (≥ 64μg/ml), imipenem (≥ 8μg/ml), meropenem (≥ 16µg/ml), doripenem (≥ 8µg/ml), amikacin (≥ 64μg/ml), gentamicin (≥ 16μg/ml), ciprofloxacin (≥4μg/ml), levofloxacin (≥8μg/ml), minocycline (≥16μg/ml), and trimethoprim/sulfamethoxazole (≥ 320µg/ml). The tigecycline MIC for BA4656 and BA34918 were 1 μg/ml and 0.06 μg/ml respectively. Based on the MIC values, we confirmed that both BA4656 and BA34918 isolates were MDR hypervirulent strains.

### General Features of the BA4656 and BA34918 Genomes

The hybrid genomes of both the isolates consisted of a chromosome assembled into a single contig along with plasmids assembled into separate circular contigs. The genome of BA4656 comprised of a 5,432,984 bp chromosome with an average 57.51% GC content and seven circular plasmids (**Supplementary Figure 1**). The genome of BA34918 consisted of a 5,439,838 bp chromosome with an average 57.5% GC content and eight circular plasmids (**Supplementary Figure 1**). The N50 values for both the hybrid assemblies (5,432,984 and 5,439,838) were found to be higher than the short-read assemblies (59,132 and 52,852) suggesting highly contiguous assemblies. In addition, the higher BUSCO values validate the completeness of the hybrid genome assembly (100%) in comparison with the short-read assembly (97.5%). As mentioned before, both genomes belong to ST23 as determined by Kleborate.

**Figure 1:**
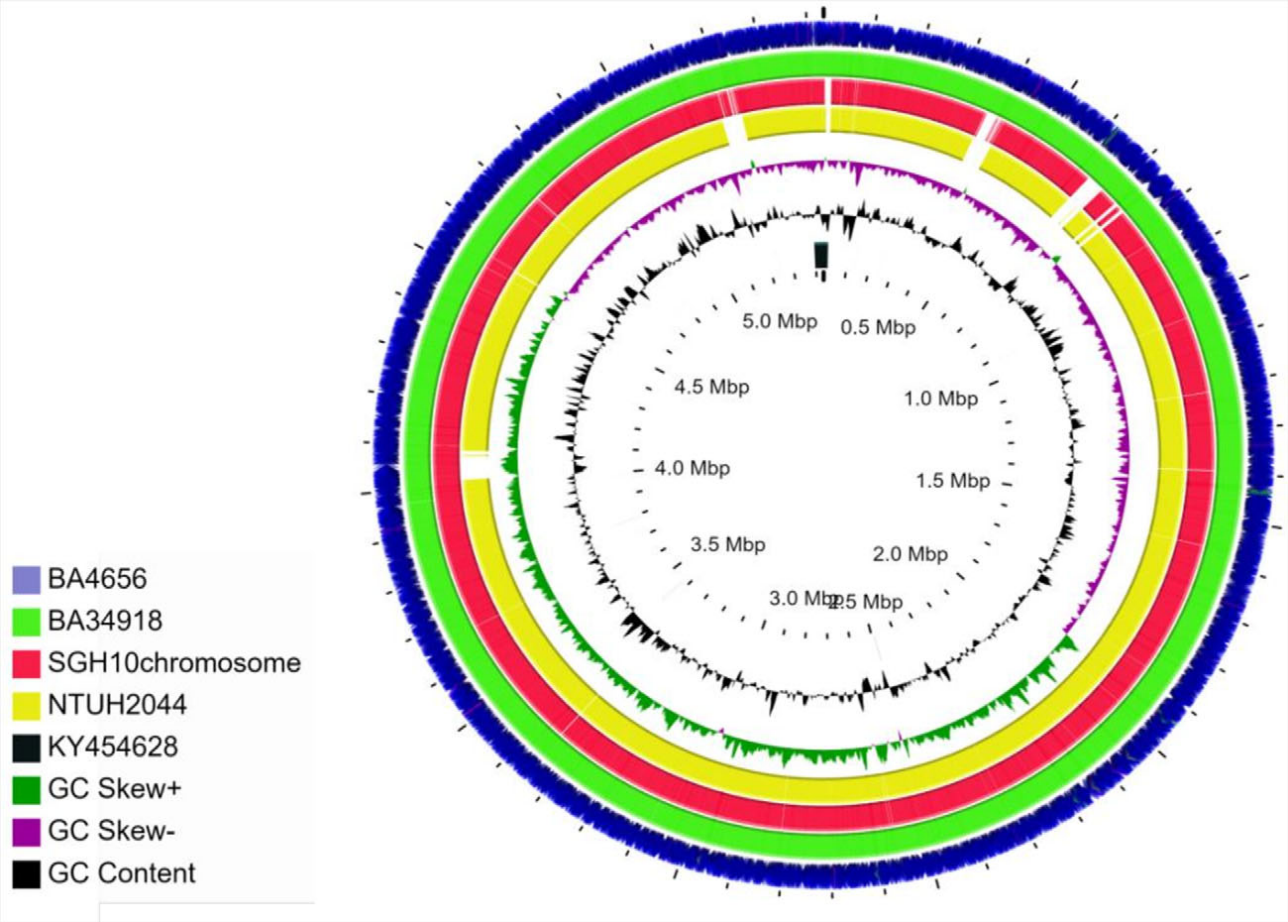
Circular representation of chromosomes of *K. pneumoniae* isolates BA4656 (NZ_CP035905) and BA34918 (NZ_CP036190) genomes displayed using CG view server with the reference genomes SGH10 (NZ_CP02580) and NTUH-K2044 (NC_012731). KY454628 is the reference used for ICEKp3. SGH10 and NTUH-2044 have ICEKp10 and ICEKp1, respectively. Genomes of BA4656 and BA34918 have numerous insertion sequences (IS) when compared to the reference genomes. These IS elements are represented as gaps in the two reference genomes.

From whole genome sequence analysis, it was found that both BA4656 and BA34918 isolates belong to ST23 with serotype K1 and O1v2. These are typical characteristics of hypervirulent *K. pneumoniae*. Comparison of the genomes of the clinical isolates characterized in this study with the two reference genomes SGH10 and NTUH2044 suggested the presence of several IS elements and other differences in the chromosomes (**Figure 1**). The two genomes in the present study were MDR and hence the presence of higher numbers of mobile genetic elements when compared to the reference genomes, which are susceptible to antimicrobials.

### Antimicrobial Resistance Among Multidrug Resistant Hypervirulent *Klebsiella pneumoniae*

The resistance phenotype of the two isolates was supported by the identification of extended spectrum β-lactamases *bla*_SHV-190_; quinolone efflux pumps *oqxA* and *oqxB*; and fosfomycin resistant gene *fos*A in the chromosome. The hybrid genome assembly also elucidated the presence of multiple MDR plasmids harboring resistance genes encoding for β-lactamases (*bla_TEM1B_*, *bla_CTX-M-15_*, *bla_CMY-4_*), aminoglycoside modifying enzymes [*aac(6’)-Ib3*, *rmtF*, *aph(6)-Id*, *aph(3)-Ib*], fluoroquinolone resistance (*qnrB1*), sulfonamide resistance (*sul1*, *sul2*), trimethoprim resistance (*dfrA14*), phenicols resistance (*catA1, catB*), and rifampicin resistance (*ARR-2*) (**Table 1**). Carbapenemase gene *bla*_OXA-232_ as well as macrolide resistance genes (*mphE, msrE*) were detected only in BA34918. The isolates carried 7–8 plasmids each and the distribution of resistance genes on the plasmids varied as mentioned in the **Table 1**.

**Table 1:** Characteristics of the two multidrug resistant (MDR) hypervirulent *Klebsiella pneumoniae* isolates belonging to ST23.

	BA4656	BA34918
Accession numbers	CP035905-CP035912	CP036190-CP036198
K type	K1	K1
O type	O1v2	O1v2
Chromosomal AMR genes	*bla*_SHV-190_, *oqxA, oqxB, fosA*	*bla*_SHV-190_, *oqxA, oqxB, fosA*
Chromosomal virulence genes	*mrkABCDFIJ, allABCDRS, glc, arcC, fdrA, fyuA, glxK, hyi, ybbW, ybbY*, KP1_1364, KP1_1371*, ylbE, ylbF*	*mrkABCDFIJ, allABCDRS, arcC, fdrA, fyuA, gcl, glxK, hyi, kfuA, kfuB, kfuC, irp2, KP1_1364, KP1_1371, ybbW, ylbE*
No. of plasmids	7	8
IncA/C_2_	*aph(6)-ld, aph(3’’)-lb*, *bla*_TEM-1B_, *bla*_CMY-4_, *bla*_CTX-M-15_, *sul2, dfrA14*	*aph(6)-ld, aph(3”)-lb, bla_CTX-M-15_ (2 copies), bla_TEM-1B_, bla_CMY-4,_ sul2, dfrA14*
IncFIIK	*aac(6’)-lb-cr*	Absent
IncFIB(pQil)	*aac(6’)-lb3, rmtF, qnrB1, ARR-2*	*rmtF, aac(6’)-lb3, qnrB1, ARR-2*
ColKP3	Absent	*bla*_OXA-232_
IncX3	No resistance genes	*aac(6’)-lb-cr, armA, msrE, mphE, sul1*
Other plasmids without AMR genes	Col440I, ColRNAI, unknown plasmid type	Col440II, ColRNAI
Yersiniabactin	Ybt9; ICEKp3	Ybt9; ICEKp3
Virulence plasmid	*catA1, rmpA2, iroB, iroC, iroD, iroN, iucA, iucB, iucC, iucD, iutA*	*catA1, rmpA, iroB, iroC, iroD, iroN, iucA, iucB, iucD, iutA*
Heavy metal resistance on virulence plasmid	*pbrA, pbrR, pcoB, pcoC, pcoD, pcoE, pcoR, pcoS, silC, silE, silR, terA, terB, terD, terE, terZ*	*pbrR, pcoA, pcoB, pcoC, pcoD, pcoE, pcoR, silS, silE, silR, terA, terB, terD, terE, terZ*

### Genomic Elements That Support Acquiring Foreign DNA

**Figure 2:**
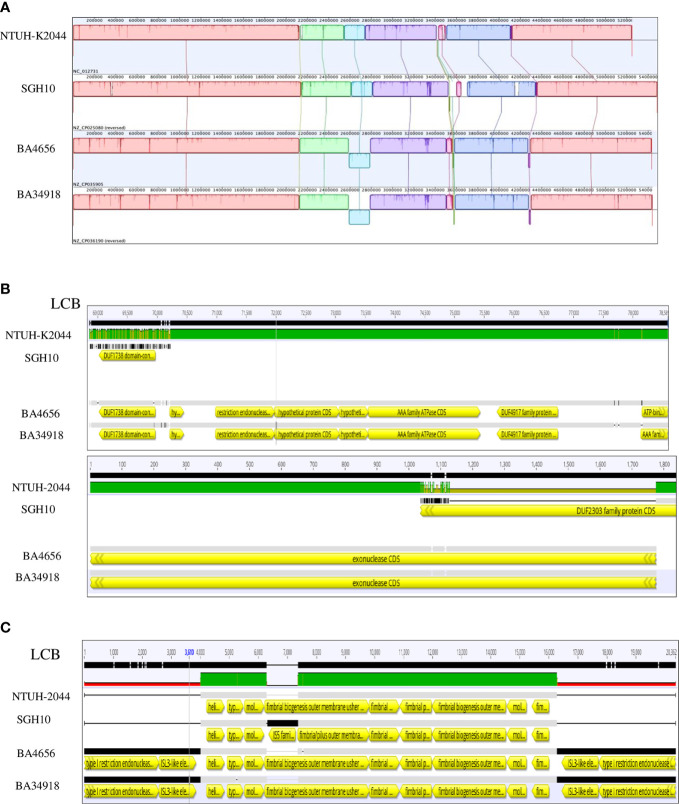
Alignment of chromosomes of *Klebsiella pneumoniae* isolates BA4656 (NZ_CP035905) and BA34918 (NZ_CP036190) with reference genomes SGH10 (NZ_CP02580) and NTUH-K2044 (NC_012731). **(A)** 12 locally co-linear blocks (LCBs) of all four strains are shown. **(B)** Presence of restriction endonucleases (LCB 6) and exonucleases (LCB 2) in the clinical isolates and not in the reference strains. **(C)** The biosynthetic operon of the *fim* gene (LCB 11) shown here is flanked by the IS3 like insertion sequence in the clinical isolates and not in the reference genomes.

Genome alignment of the two isolates along with two reference genomes used in this study shows several areas in the genome that differ among the strains (**Figures 1** and **2**). The genome alignment has identified the presence of genes in the clinical isolates that are putatively part of restriction modification systems, which are absent in the two reference strains (**Figures 2B, C**). A total of 15 different TA systems in *K. pneumoniae* genomes were identified in all the four strains (including two references). Among the 15 TA systems, only 11 were common in all the strains that include all the major families. The study isolates BA34918 and BA4656 encode 11 and 14 TA systems respectively (**Table 2**).

**Table 2:** Chromosomal toxin and antitoxin systems encoded by *Klebsiella pneumoniae*.

NTUH-K2044	SGH10	BA4656	BA34918
HipA-like (T)	HipA-like (T)	HipA-like (T)	HipA-like (T)
Xre-like (A)	Xre-like (A)	Xre-like (A)	Xre-like (A)
RelE-like (T)	RelE-like (T)	RelE-like (T)	RelE-like (T)
Xre-like (A)	Xre-like (A)	Xre-like (A)	Xre-like (A)
GNAT-like (T)	GNAT-like (T)	GNAT-like (T)	GNAT-like (T)
RHH-like (A)	RHH-like (A)	RHH-like (A)	RHH-like (A)
RelE-like (T)	RelE-like (T)	RelE-like (T)	RelE-like (T)
RHH-like (A)	RHH-like (A)	RHH-like (A)	RHH-like (A)
PIN-like (T)	PIN-like (T)	PIN-like (T)	PIN-like (T)
RHH-like (A)	RHH-like (A)	RHH-like (A)	RHH-like (A)
Fic-like (T)	RelE-like (T)	Fic-like (T)	Fic-like (T)
PHD-like (A)	Doc (A)	PHD-like (A)	PHD-like (A)
pfam12568 (T)	pfam12568 (T)	pfam12568 (T)	pfam12568 (T)
pfam00392 (A)	pfam00392 (A)	pfam00392 (A)	pfam00392 (A)
pfam12568 (T)	pfam12568 (T)	pfam12568 (T)	pfam12568 (T)
pfam01047 (A)	pfam01047 (A)	pfam01047 (A)	pfam01047 (A)
pfam12568 (T)	pfam12568 (T)	pfam12568 (T)	pfam12568 (T)
cd00093 (A)	cd00093 (A)	cd00093 (A)	cd00093 (A)
COG1246 (T)	COG1246 (T)	COG1246 (T)	COG1246 (T)
pfam00392 (A)	pfam00392 (A)	pfam0568 (A)	pfam00392 (A)
Fic-like (T)	Fic-like (T)	Fic-like (T)	Fic-like (T)
YhfG-like (A)	YhfG-like (A)	YhfG-like (A)	YhfG-like (A)
GNAT-like (T)	GNAT-like (T)	GNAT-like (T)	Absent
RHH-like (A)	RHH-like (A)	RHH-like (A)	Absent
RelE-like (T)	RelE-like (T)	RelE-like (T)	Absent
Xre-like (A)	Xre-like (A)	Xre-like (A)	Absent
pfam01325 (T)	Absent	pfam01325 (T)	Absent
pfam12568 (A)	Absent	pfam0568 (A)	Absent
PIN-like (T)	Absent	Absent	Absent
AbrB-like (A)	Absent	Absent	Absent

T, toxin; A, antitoxin. The length of toxin proteins varied between 94 and 465 amino acids, while the length of the antitoxin proteins varied between 55 and 319 amino acids.

### Mobile Genetic Elements

#### Plasmids and Transposons

**Figure 3:**
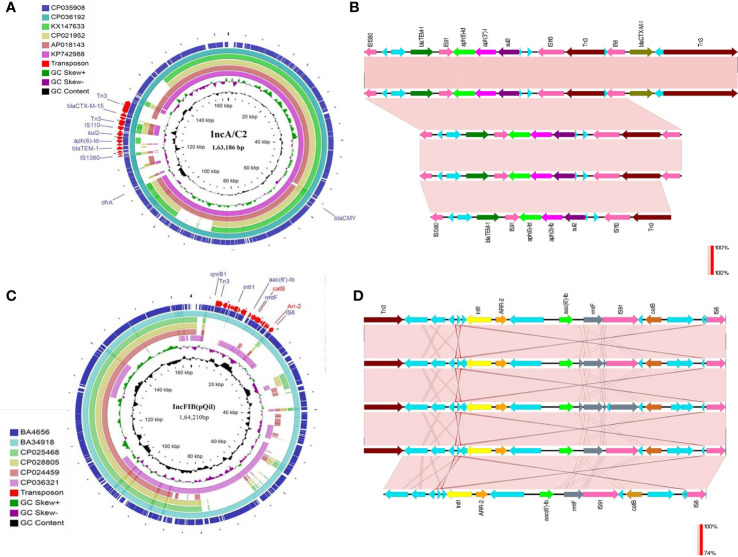
**(A)** The structures of the IncA/C_2_ plasmids identified in the present study (CP035908, 163,186bp and CP036192, 165,052bp). Sequence comparison with other IncA/C_2_ plasmids showed that the plasmid displayed 99% conservation with IncA/C_2_ plasmids from *Klebsiella pneumoniae* AR_0148 (CP021952), *Citrobacter freundii* (KX147633), *Escherichia coli* (AP018143), and *Salmonella* Senftenberg (KP742988). Tn6691 on IncA/C_2_ carried *bla*_TEM-1B_, *bla*_CTX-M-15_, *aph(6)-ld* and *aph(3’’)-lb*. **(B)** Comparison of the *Tn6691* elements on IncA/C_2_ among B4656 (CP035908) and BA34918 (CP036192) with *E. coli* strain 109 (CP020523), *K. pneumoniae* strain INF249 (CP024489), and *Salmonella enterica* subsp. *enterica* serovar *Typhimurium* strain UGA14 (CP021463) in the above-mentioned order. **(C)** The structures of the IncFIB (pQil) plasmids identified in the present study (CP035907, 164,210bp and CP036194, 108,957bp). Tn*6692* present on IncFIB(pQil) carried *ARR-2*, *catB*, *rmtF*, and *aac(6’)-lb3*. **(D)** Comparison of the transposon Tn*6692* on IncFIB in the following order: *K. pneumoniae* strains AR0138 (CP021758), BA4656 (CP035907), BA34918 (CP036193), KPN1482 (CP020842), and SKGH01 (CP015500).

Both the isolates harbored six common putative distinct plasmid replicons; namely IncA/C_2_, IncFIB (pQil), IncFIB, IncX_3_, ColRNAI, and Col440II along with a common virulence plasmid (**Supplementary Figure 1**). Surprisingly, the isolate BA34918 carried an additional ColKP3 non-conjugative plasmid that harbored the carbapenemase gene *bla_OXA-232_* (CP036197). The presence of such a large number of plasmids among the ST23 sequence types has not been previously documented. Among the groups, the IncA/C_2_ plasmid is particularly important as it encodes multiple determinants (**Table 1**). These resistance genes were inserted within the transposon Tn*6691*, in the IncA/C_2_ plasmid (**Figure 3**). Sequence comparison with other IncA/C_2_ plasmid showed that the plasmid displayed 99% conservation with IncA/C_2_ plasmids from *K. pneumoniae* AR_0148 (CP021952), *Citrobacter freundii* (KX147633), *E. coli* (AP018143), and *Salmonella* Senftenberg (KP742988) (**Figure 3**). Considering the broad bacterial host range of IncA/C_2_ plasmid, acquisition of these plasmids contributed to the evolution of MDR hvKp from pan susceptible hvKp.

Similarly both the isolates harbored resistance genes *qnrB1*, *catB*, *aac(6’)-Ib3*, *rmtF*, and *ARR-2* within a novel transposon Tn*6692*, on the IncFIB(pQil) plasmid (**Figure 3**). Although both BA4656 and BA34918 strains were found to harbor IncX3 plasmid, BA4656 lacked the resistance cassette *aac(6’)-Ib-cr, armA, mphE, msrE*, and *sul-1* that is inserted into the IncX3 backbone of BA34918 (CP036195). The other two small ColRNAI (9.729 bp) and Col440II (4.166 bp) plasmid replicons does not carry any resistance genes or mobile genetic elements.

#### Virulence Plasmid

**Figure 4:**
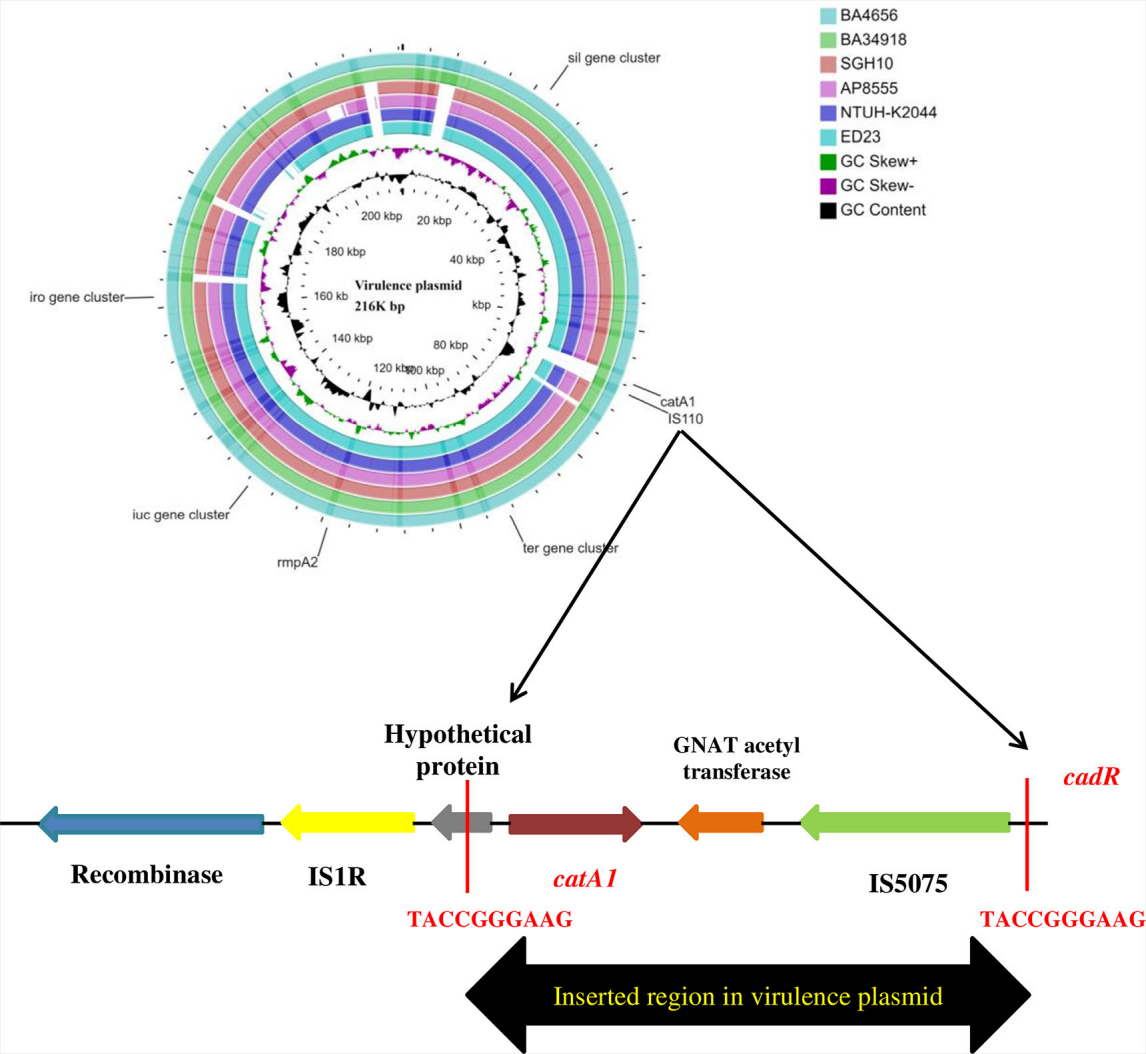
Comparison of virulence plasmid of hypervirulent (hv) *Klebsiella pneumoniae* isolates BA4656 and BA34918 with virulence plasmids from *K. pneumoniae* strains SGH10, NTUH-K2044, AP8555, and ED23. The plasmid carried virulence genes including *rmpA/rmpA2*, *iro*, and *iuc* gene clusters along with heavy metal resistance encoding *ter, sil*, and *pco* gene clusters. *catA1*, coding for chloramphenicol resistance with IS5075 (IS110 family) was inserted in plasmids of BA4656 and BA34918. Inserted region of 2,744 bp in the virulence plasmid carrying *catA1* is shown below. Inserted region is flanked by a repeat region of 10bp (TACCGGGAAG) which could possibly be a hotspot for integration.

The large virulence plasmid (2,16,620 bp) of the two hvKp isolates were highly similar to the pLVPK-like virulence plasmid with < 50% sequence coverage. The BLAST based identity search for the homologous sequences showed similarity with well characterized virulence plasmids from other CC23 isolates such as *K. pneumoniae* strains NTUH-K2044 (AP006726; 99.63%), ED23 (CP016815; 99.63%), and SGH10 (CP025081; 99.62%). The comparison of the virulence plasmid of both the isolates with the reference virulence plasmid is shown in **Figure 4**. Surprisingly, we found that the virulence plasmid of both BA4656 and BA34918 isolates encode a chloramphenicol resistance gene (*catA1*), which is probably inserted through IS110 family transposase. The inserted region comprised of 2,744bp flanked by 10 bases of terminal repeat (TACCGGGAAG) and this was inserted between a hypothetical protein and IS5075 belonging to the IS110 family (**Figure 4**). The insertion of a resistance gene into the virulence plasmid indicates a potential hotspot for the further acquisition of other resistance genes into the virulence plasmid.

The phenotypic features encoded by the virulence plasmids (CP035906 and CP036191) included the salmochelin (*iroBCDN*) and aerobactin (*iucABCDiutA*) clusters which help in utilizing iron from the host (Shon et al., 2013). The presence of *rmpA* and *rmpA2* along with K1 capsule type confirms a hypermucoid phenotype that is a characteristic feature of hypervirulent isolates (Shon et al., 2013). The virulence plasmids also code for resistance to heavy metals such as copper, lead, silver, and tellurite (**Table 1**). This contributes toward resistance to some disinfectants and helps hvKp thrive in hospital settings.

In addition to the virulence genes encoded by the plasmid, in *K. pneumoniae* the chromosome also codes for virulence factors. Chromosomal virulence factors include type3 fimbria coded by *mrk* operon (*mrkABCDFIJ)*, allantoin regulation (*allABCDRS*), and *kfu* operon encoding iron uptake system (*kfuABC*) which contribute to the high virulence and invasiveness of the isolates. In addition, *ybbW, ybbY glc, fdrA, glxK, arc*, and *hyi* are other genes coding for iron uptake (**Table 1**).

### Genomic Island

**Figure 5:**
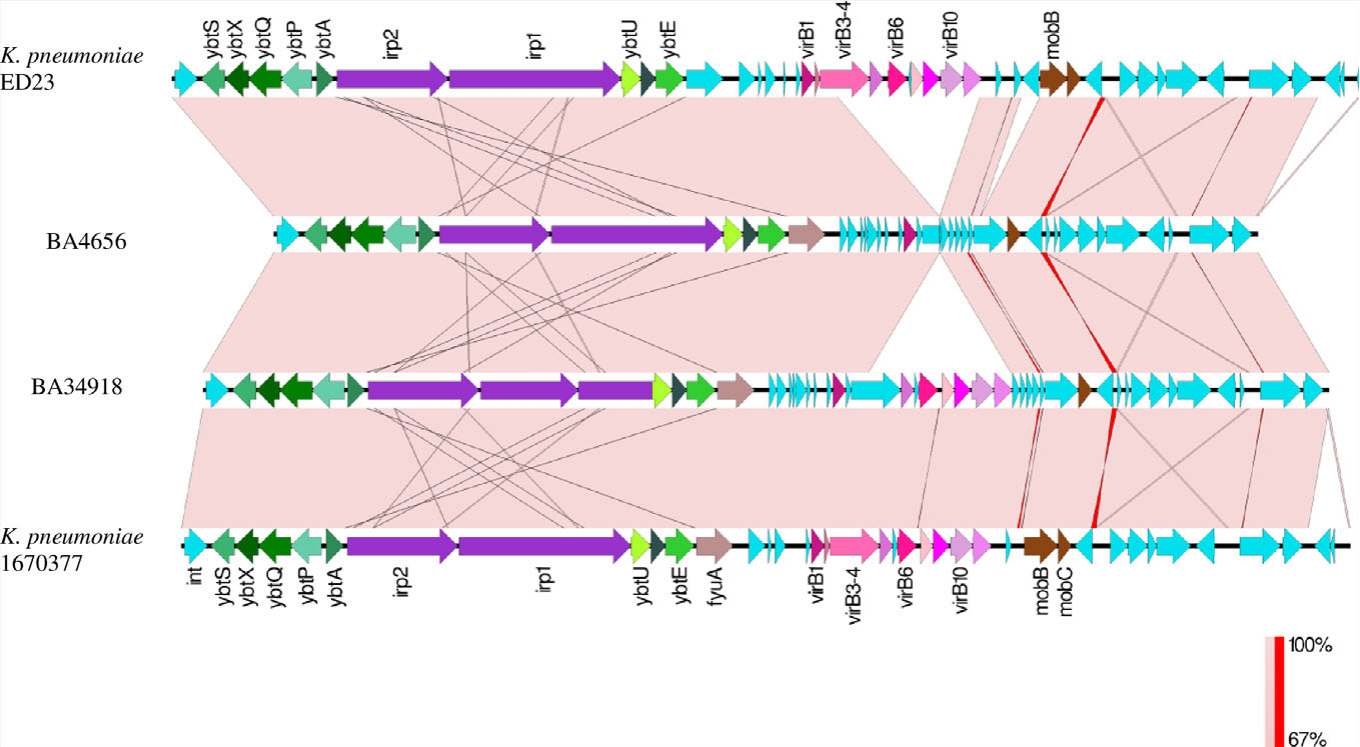
Comparison between ICEKp3 genomic islands (GI) of hypervirulent (hv) *Klebsiella pneumoniae* isolates BA4656 and BA34918 with ICEKp3 GI from other *K. pneumoniae* strains ED23 and 1670377. This island contains the yersiniabactin gene cluster and the type IV secretion system (T4SS) *virB* gene cluster. ICEKp3 of BA4656 was shorter than BA34918 since it lacked some of the T4SS genes.

In hvKp, the yersiniabactin locus (*ybtAPSTUX*) was located within diverse Integrative congregative elements (ICE*Kp*) present on the chromosome. Both the isolates carried the yersiniabactin locus *ybt9* located in ICE*Kp*3. The diversity of the detected ICE*Kp*3 was analyzed by mapping against ICE*Kp* regions from isolates ED23 (CP016814) and the *K. pneumoniae* strain 1670377 (KY454628) available in GenBank (**Figure 5**). Other than the yersiniabactin locus, the characteristic virulence factors of ICE*Kp* including the siderophore genes *irp1* and *irp2*, the ferric yersiniabactin uptake gene *fyuA*, the virB-type 4 secretion system (T4SS), and mobBC (mobilization) proteins were also identified. Another interesting observation is the *fim* operon, a genomic region encoding a major virulence factor, is flanked by insertion sequence elements only in the clinical isolates (**Figure 2C**). This indicates that the *fim* genes might have been recently acquired by the clinical strains as a selective advantage in infecting host tissues.

### Phylogenetic Analysis

The two hvKp isolates (BA4656 and BA34918), were compared with the genome sequences of 187 global CG23 isolates and 5 pan-susceptible Indian ST23 isolates. Phylogenetic analysis revealed a number of sub-lineages with the globally distributed CG23-I (CG23 sub-lineage I) comprising of 158 isolates. The study isolates BA4656 and BA34918 formed a separate sub-lineage (referred as sub-lineage II hereafter) with isolates from India, Southeast Asia, China, and Europe. An earlier pan-susceptible isolate from the same study center (BA253) as well as another isolate of Indian origin (VINI01) was also clustered with the sub-lineage II. The observed median pairwise SNP distance after removing the recombinant events was 191 SNPs (range 1–719 SNPs). Further the median pairwise SNP distance between CG23-I and sub-lineage II was identified as 307 SNPs (range 122–719 SNPs). The closest isolate (VINI01) differed from BA4656 and BA34918 by 311 and 328 SNPs respectively whereas the differences between the two study isolates were 453 SNPs.

**Figure 6:**
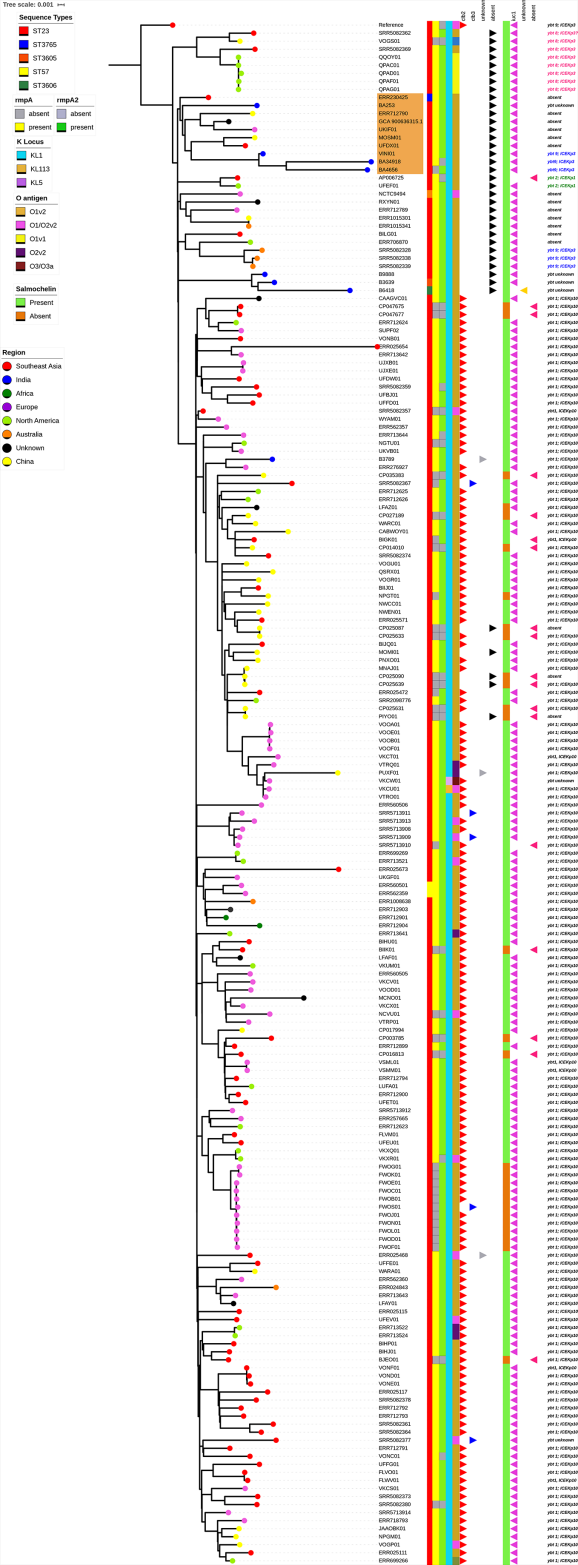
Single nucleotide polymorphism (SNP) based phylogenetic tree based on the core genome alignment of 192 CG23 *Klebsiella* *pneumoniae* isolates from the global collection. The maximum likelihood phylogram is based on 9,300 SNPs after removing the recombinant regions. The phylogenetic tree was rooted by using the reference genome (ED23). The scale represents the evolutionary distances. The percentage of 100 bootstrap trials (100 replicates). The study isolates BA4656 and BA34918 formed a separate sub-lineage with 10 isolates distributed around the world in the phylogenetic tree (highlighted in orange color).

The sub-lineage II either lacked yersiniabactin or carried *ybt9* on ICEKp3 (**Figure 6**). Among the global collection, 153 (79.6%) isolates that belong to CG23-I were characteristically associated with *ybt1* carried on ICEKp10 while other sub-lineages either lack ICEKp or carry *ybt8/9* on ICEKp3. Further, isolates belonging to sub-lineage II produced aerobactin and salmochelin but lacked colibactin. Notably, all the 192 isolates belonged to K1 capsule type except for two European isolates. In contrast, six diverse types of O antigen were observed with O1v2 being the most common in 165 (86%) isolates. Also, 20 isolates predominantly from Asian countries, lacked *rmpA* and *rmpA2*.

## Discussion

Though our recent studies have indicated the characterization of hvKp from India (Shankar et al., 2016a; Shankar et al., 2018), the clinical, microbiological, and evolutionary features of MDR hvKp from India remain largely unknown. Both the isolates characterized in this study possessed the hypermucoviscous phenotype and the large virulence plasmid which has >90% similarity with the large virulence plasmid pLVPK of *K. pneumoniae* strain NTUH-K2044. Our results indicate the coexistence of multiple resistance plasmids such as IncA/C_2_, IncFIB, IncX3, and ColKP3 along with the virulence plasmid within the same hvKp isolate. Interestingly, these MDR plasmids are known to play an important role in the dissemination of antimicrobial resistance in *K. pneumoniae* worldwide (Martin and Bachman, 2018). These multiple acquisitions of MDR plasmids are surprising and unprecedented for hvKp as the clone is generally less likely to acquire multiple MDR plasmids (Wyres et al., 2019). However, due to antibiotic selection pressure hvKp appears to be acquiring multiple MDR plasmids leading to MDR-hvKp (Chen et al., 2020; Zhang et al., 2020).

The increasing genetic plasticity of hvKp has been reported from multiple geographical locations (Cejas et al., 2014; Zhang et al., 2016; Xie et al., 2018; Zhang et al., 2020). Moreover, several studies from other Asian countries have documented the predominant carbapenem resistance *K. pneumoniae* (CRKp) clones acquiring virulence plasmid from hvKp (Gu et al., 2018). Therefore, plasmid exchange appears to be happening both ways between hvKp and the prevalent sequence type of CRKp in study setting. We found that in Indian settings the possible exchange of hvKp and CRKp plasmids might have occurred between the hvKp clone ST23 and the CRKp clone ST231. Although the direct proof of exchange is absent, we speculate that ST231 *K. pneumoniae* is the most prevalent sequence type of CRKp in the same setting (Shankar et al., 2019). Given the high frequency and the number of MDR plasmids acquired, the MDR hvKp BA4656 and BA34918 can be considered as a “real superbug” and it might have serious implications to public health.

CC23 persisters showed higher survival rate at a dose of 10x MIC against a carbapenem class antibiotic meropenem (Lee et al., 2019). This indicates clinical isolates, belonging to CC23 complex, used in this study may be capable of forming persisters under high dose of carbapenem and thus can find opportunity to acquire resistance genes from the pool of dead bacteria. This, in turn, could allow for active growth in the presence of antibiotics from the once dormant persister bacteria. This is alarming because carbapenems are last resort to treat *K. pneumoniae* infection (Papp-Wallace et al., 2011). Since TA systems are the major players of persister formation, we investigated the presence of TA systems in the two study isolates (**Table 2**). Among the three additional TA systems present in the clinical isolates, one was uniquely present in the plasmid, while the other two were also present in the chromosome. In general, TA systems are also involved in diverse cellular processes. Plasmid maintenance, selfish alleles, gene regulation, growth control, persister formation, programmed cell arrest and death, anti-phage activity, biofilm formation, and general stress response can all be controlled by TAs (Ramage et al., 2009).

Among the multiple plasmids acquired by our isolates, IncA/C_2_, IncFIB, and ColKP3 are of particular importance. Notably the acquisition of the IncA/C_2_ and the IncFIB plasmids is considered to be responsible for the spread of resistance among Enterobacterales (Rozwandowicz et al., 2018). The nucleotide sequence similarity of plasmids in other Enterobacterales such as *Citrobacter* sp. (KX147633) *E. coli* (AP018143) and *Salmonella* sp. (KP742988) substantiates the horizontal dissemination of plasmid among different bacterial species. The Tn*6691* transposon located on the IncA/C_2_ plasmid has been found to be responsible for the dissemination of AMR genes such as *bla*_TEM-1B_, *bla*_CTX-M-15_, *aph(6)-ld*, and *aph(3’’)-lb.* Similarly IncFIB, which was present in both the isolates, contributes to increased resistance to antimicrobials by carrying AMR genes such as *aac(6’)-lb3, rmtF, qnrB1, ARR-2*. The acquisition of the *bla*_OXA-232_-bearing ColKP3 plasmid by a single isolate of hvKp may mark a major evolutionary step toward further establishment of clonal complex 23 (CC23). Till now, the clonal dissemination of *bla*_OXA-232_-bearing *K. pneumoniae* majorly occurred by means of the ST231 carrying ColKP3 plasmid in India (Shankar et al., 2019). The emergence of carbapenemase producing hvKp in India can be hypothesized to be due to acquiring the ColKP3 plasmid from ST231 CRKp, as this sequence type is widely circulating in the same environment (Shankar et al., 2019). Thus, the emergence of *bla*_OXA-232_ producing hvKp is particularly concerning due to its combination of hypervirulence and carbapenem resistance.

Plasmids such as IncX3, ColKP3, IncA/C_2_ are common among members of Enterobacterales and are responsible of dissemination of antimicrobial resistance (AMR). **Supplementary Figures 2**–**4** shows the comparison of these plasmids isolated from *K. pneumoniae* and *E. coli* from the study centre. Though similar AMR genes are mobilized by these plasmids in both the organisms, significant differences were observed among ColKP3. Two ColKP3 plasmids from *E. coli* carried *bla*_OXA-181_ and were 50% similar to *K. pneumoniae* ColKP3. IncX3 was highly similar among both the organisms. In the present study, IncFIB (pQil) and IncA/C_2_ that were present in *K. pneumoniae* were not identified among the *E. coli* in this study collection. Hence, from global database, representative IncA/C_2_ plasmids were used to construct a phylogenetic tree (**Supplementary Figure 4**). It was observed that IncA/C_2_ commonly coded for aminoglycoside and β-lactam resistance genes. *bla*_NDM_ was the most common gene disseminated by this plasmid.

The characteristic feature of hvKp is the presence of the 216 kb large virulence plasmid with the mucoid regulators *rmpA* and *rmpA2* being encoded in the plasmid**.** In addition, the virulence plasmid is characterized by the presence of the *catA1* gene flanked by the insertion elements IS1 and IS110 suggesting that the region has been inserted into the virulence plasmid (Mahillon and Chandler, 1998). A similar recombination event in the same virulence plasmid was previously reported by Dong and colleagues (2018), where a *bla*_KPC-2_ carrying region was inserted into the virulence plasmid. Conversely there have been reports of the acquisition of a hvKp virulence plasmid by CRKp isolates (Gu et al., 2018). Notably, the emergence of a MDR hvKp carrying hybrid/fusion plasmid by the integration of a fragment of the hypervirulence plasmid into a MDR plasmid suggests the constant evolution of hvKp clonal lineages (Lam et al., 2019).

The SNP based phylogeny of the two isolates with respect to the representative CG23 global strains showed that the evolutionary events are not driven by geographical location. The phylogenetic distribution indicated distinct sub-lineages with a major sub-lineage (CG23-I) associated with liver abscess strains (Lam M. et al., 2018). This observation is in line with Lam and colleagues (2018) where this globally distributed sub-lineage is driven by ICEKp10 with *ybt1.* The sub-lineage II where two study isolates clustered were predominantly associated with sepsis and other invasive infection. However, the evolution of sub-lineage II is not consistent with ICEKp distribution.

In addition, when growth of the two study isolates was compared with ST231 *K. pneumoniae* from the same center, at 24 h, the isolate BA34918 and the ST231 Kp showed higher growth when compared to BA4656 (**Supplementary Figure 5**). However, after 25h, the ST231 Kp showed a rapid decline in growth while no decline in the growth of two hvKp was observed. The acquisition of antimicrobial resistance including colistin resistance in hvKp has been associated with increased fitness cost (Choi and Ko, 2015; Lee et al., 2017). Correspondingly, the ST23 hvKp with *bla*_OXA-232_ in the present study showed increased fitness when compared to the isolate without *bla*_OXA-232_.

Based on the genetic composition of the virulence plasmids, which had multiple insertion elements, we speculate further plasmid recombination events will occur leading to emergence of novel resistance-virulence encoding plasmids. Our findings are relevant in understanding the future risk of the emergence of individual *K*. *pneumoniae* strains carrying both the virulence and acquired resistance genes, capable of causing highly virulent infections, which will be extremely difficult to control. Specifically, our data indicate that MDR clones pose the greatest risk because they are more likely to acquire virulence genes than the hypervirulent clones acquiring resistance genes.

## Data Availability Statement

The accession numbers of the genomes deposited in GenBank, NCBI, are mentioned in methods and **Table 1**.

## Ethics statement

The study was reviewed and approved by Institutional Review Board, Christian Medical College and Hospital, Vellore with IRB min no. 9616 dated 1st September 2015. Written informed consent for participation was not required for this study since the bacterial isolates were used without patient identifier.

## Author contributions

CS: Laboratory methods, data analysis and interpretation, manuscript writing. JJ: Data analysis, interpretation and manuscript writing. KV: Hybrid genome assembly and other bioinformatics methods. RB: Data analysis, manuscript writing. DPMS: Data analysis. AB: Clinical details and expert opinion on study design. SV: Clinical details and expert opinion on study design. IB: Manuscript correction. BV: Study design and supervising, manuscript writing, manuscript correction. All authors contributed to the article and approved the submitted version.

## Conflict of Interest

The authors declare that the research was conducted in the absence of any commercial or financial relationships that could be construed as a potential conflict of interest.
