# Supplementary material for: Emergence of Multidrug Resistant Hypervirulent ST23 Klebsiella pneumoniae: Multidrug Resistant Plasmid Acquisition Drives Evolution
Source: Front Cell Infect Microbiol. 2020 Nov 20;10:575289. doi: 10.3389/fcimb.2020.575289 (PMC7718023; doi:10.3389/fcimb.2020.575289)
Supplement: Supplementary file 1 [file DataSheet_1.docx]

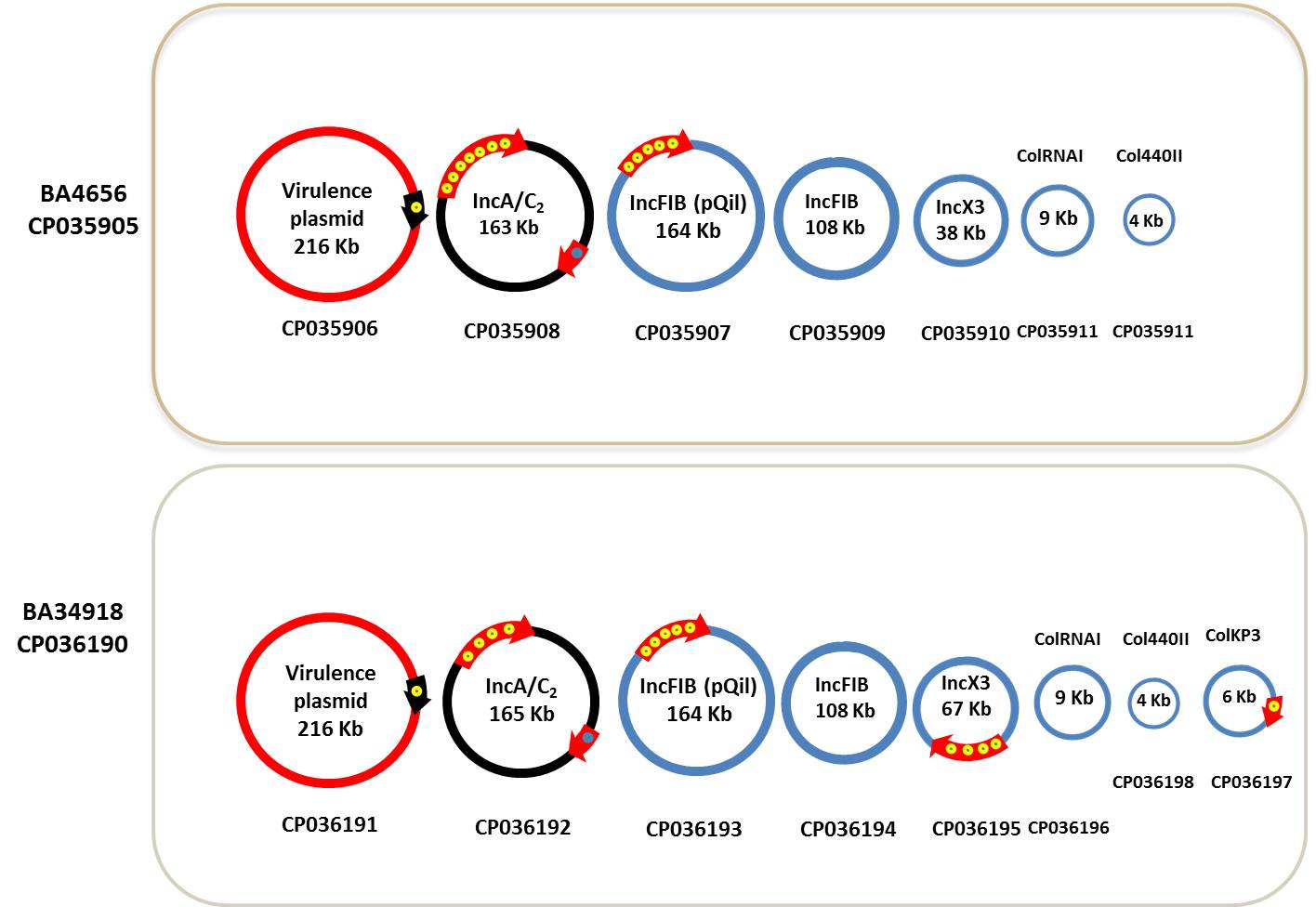
**Supplementary Figure1:** Plasmid profile of MDR hv *K. pneumoniae* isolates BA4656 and BA34918. Yellow circles indicate the presence of AMR genes


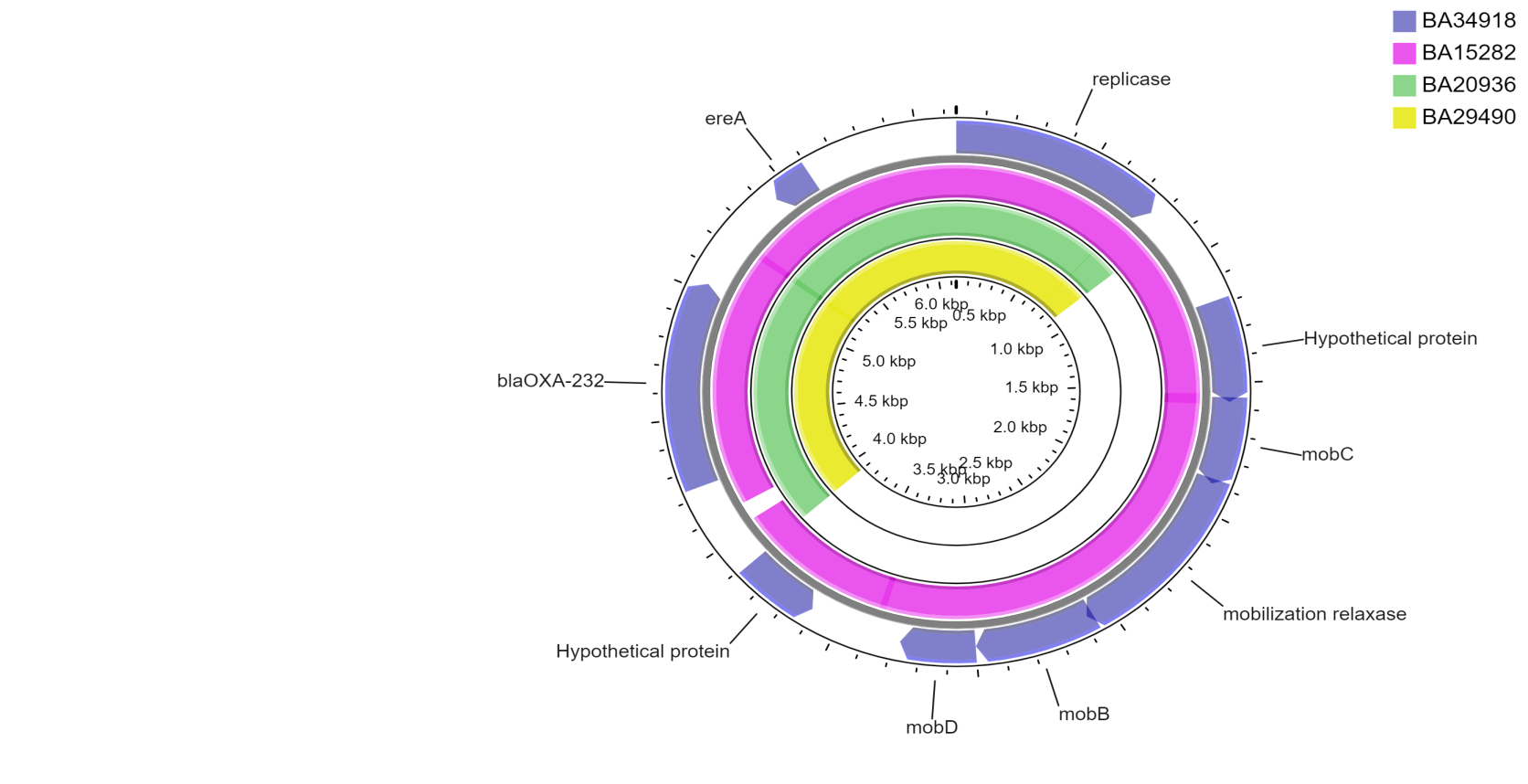


**Supplementary Figure2: Comparison of ColKp3 plasmids among *K. pneumoniae* and *E. coli* from India**

BA34918 belongs to *K. pneumoniae* while the other three are from *E. coli*. The plasmid from *E. coli* BA15282 is ~98% similar to that of *K. pneumoniae* ColKp3. In contrast, ColKp3 from BA20936 and BA29490 were 50% similar to ColKp3 of BA34918. The plasmids from BA20936 (94K bp) and BA29490 (52K bp) were large and carried IncX3 *repA* gene as well. Also, these two large plasmids carried *bla*_OXA-181_ unlike the *K. pneumoniae* ColKp3 which carried *bla*_OXA-232_.


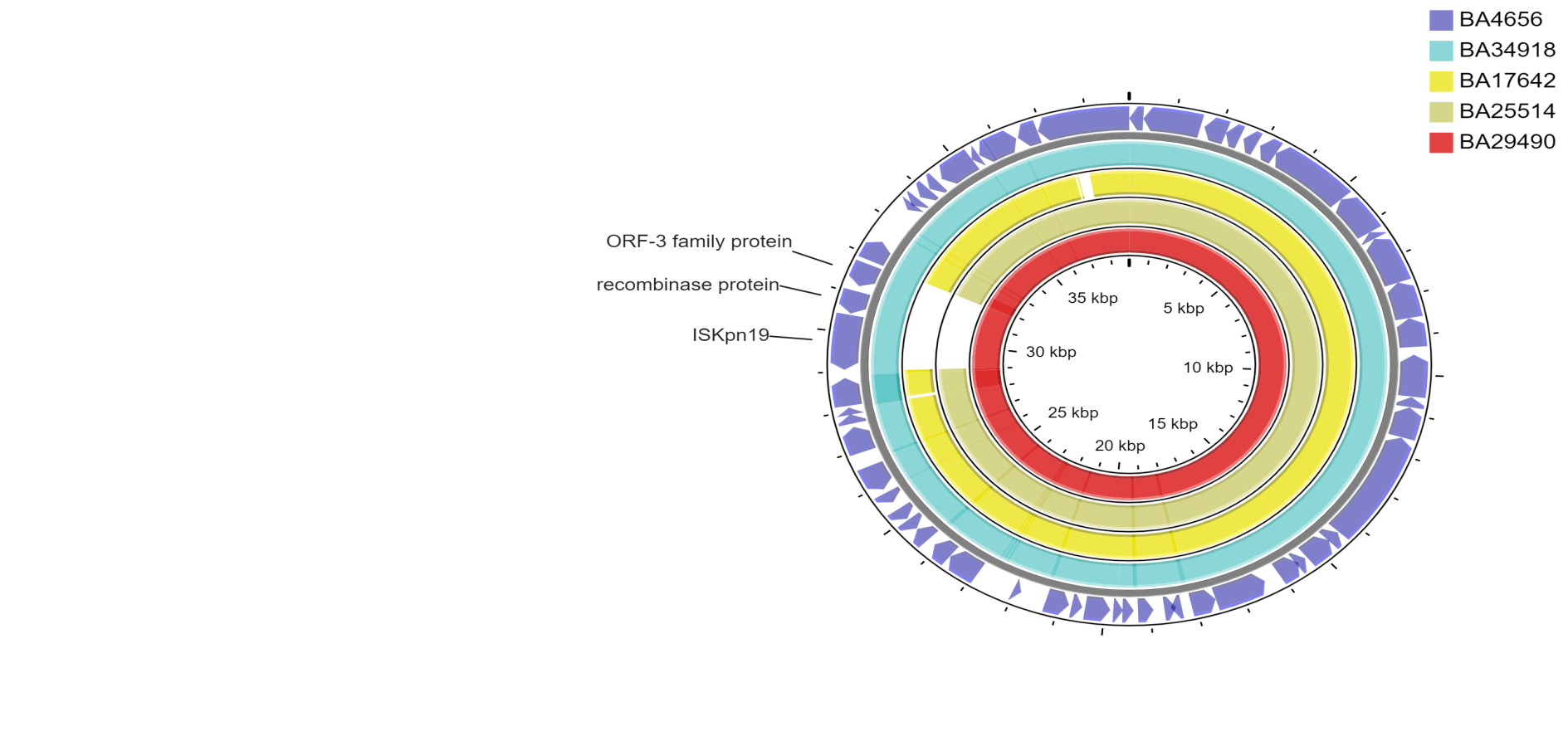


**Supplementary Figure3: Comparison of IncX3 plasmids among *K. pneumoniae* and *E. coli* from India**

Plasmids BA4656 and BA34918 were from *K. pneumoniae* while the other three were IncX3 obtained from *E. coli*. IncX3 of BA29490 (*E. coli*) is 100% identical to the IncX3 from *K. pneumoniae*. Two plasmids from *E. coli*, BA17642 and BA25514, were >95% similar to *K. pneumoniae* plasmids but lacked three genes such as ORF-3 family protein, recombinase protein and IS*Kpn19*.

**
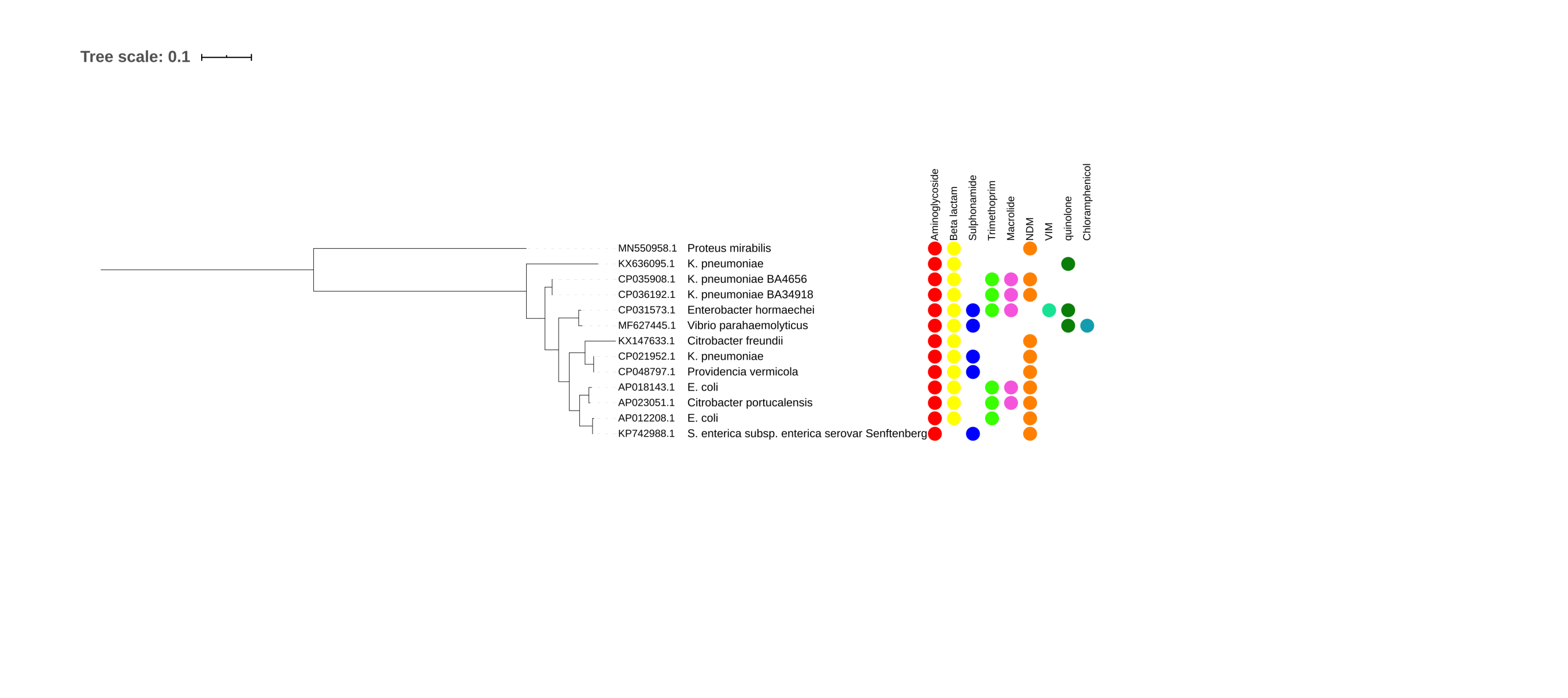

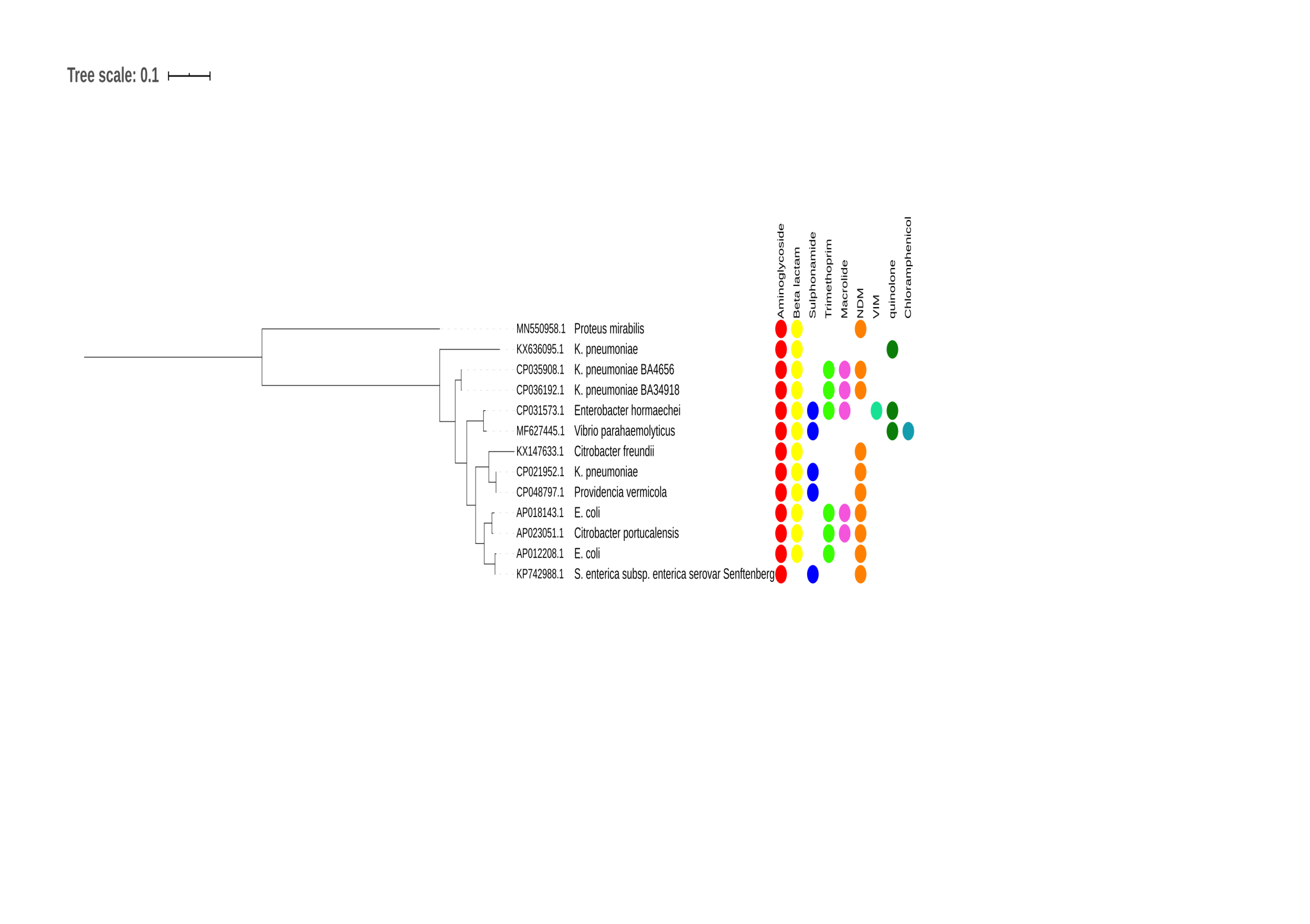
**

**Supplementary Figure4: Phylogenetic tree of IncA/C2 plasmids obtained from various Enterobacterales**

IncA/C2 plasmids from various organisms obtained from NCBI were used to construct a phylogenetic tree. All the IncA/C2 plasmids carried *bla*_CMY_ and at least one gene encoding aminoglycoside resistance. *bla*_NDM-1_ was another gene commonly disseminated by IncA/C2. The plasmids identified in the present study (CP035908 and CP036192) were closely related to IncA/C2 from another *K. pneumoniae* (KX636095), *E. hormaechei* (CP031573) and *V. parahaemolyticus* (MF627445).

Time in hours

O.D value at 530nnm

**Supplementary Figure5: Growth curve of ST23 *K. pneumoniae* when compared to ST231 *K. pneumoniae***

BA4656 and BA34918 belonging to ST23 and BA36749 belongs to ST231. O.D. value was measured at 530nm for the cultures grown in Luria Bertani broth and BA34918 showed higher growth when compared to other two isolates. BA36749 showed higher growth when compared BA4656. BA34918 and BA36749 carried *bla*_OXA-232_ on ColKp3. The higher growth in these two isolates can also be attributed to the presence of carbapenemase which might contribute to higher fitness.
